# Multiple climate change-driven tipping points for coastal systems

**DOI:** 10.1038/s41598-021-94942-7

**Published:** 2021-07-30

**Authors:** Patrick L. Barnard, Jenifer E. Dugan, Henry M. Page, Nathan J. Wood, Juliette A. Finzi Hart, Daniel R. Cayan, Li H. Erikson, David M. Hubbard, Monique R. Myers, John M. Melack, Sam F. Iacobellis

**Affiliations:** 1 Pacific Coastal and Marine Science Center, U.S. Geological Survey, Santa Cruz, CA 95060 USA; 2 Marine Science Institute, University of California, Santa Barbara, Santa Barbara, CA 93106 USA; 3 U.S. Geological Survey, Western Geographic Science Center, Portland, OR 97201 USA; 4 Scripps Institution of Oceanography, University of California, San Diego, La Jolla, CA 92037 USA; 5 California Sea Grant, University of California, Santa Barbara, Santa Barbara, CA 93106 USA; 6 Department of Ecology, Evolution, and Marine Biology, University of California, Santa Barbara, Santa Barbara, CA 93106 USA

**Keywords:** Climate sciences, Ocean sciences, Physical oceanography, Climate-change adaptation, Climate-change impacts, Climate-change mitigation, Climate-change policy

## Abstract

As the climate evolves over the next century, the interaction of accelerating sea level rise (SLR) and storms, combined with confining development and infrastructure, will place greater stresses on physical, ecological, and human systems along the ocean-land margin. Many of these valued coastal systems could reach “tipping points,” at which hazard exposure substantially increases and threatens the present-day form, function, and viability of communities, infrastructure, and ecosystems. Determining the timing and nature of these tipping points is essential for effective climate adaptation planning. Here we present a multidisciplinary case study from Santa Barbara, California (USA), to identify potential climate change-related tipping points for various coastal systems. This study integrates numerical and statistical models of the climate, ocean water levels, beach and cliff evolution, and two soft sediment ecosystems, sandy beaches and tidal wetlands. We find that tipping points for beaches and wetlands could be reached with just 0.25 m or less of SLR (~ 2050), with > 50% subsequent habitat loss that would degrade overall biodiversity and ecosystem function. In contrast, the largest projected changes in socioeconomic exposure to flooding for five communities in this region are not anticipated until SLR exceeds 0.75 m for daily flooding and 1.5 m for storm-driven flooding (~ 2100 or later). These changes are less acute relative to community totals and do not qualify as tipping points given the adaptive capacity of communities. Nonetheless, the natural and human built systems are interconnected such that the loss of natural system function could negatively impact the quality of life of residents and disrupt the local economy, resulting in indirect socioeconomic impacts long before built infrastructure is directly impacted by flooding.

## Introduction

A tipping point “refers to a critical threshold at which a tiny perturbation can qualitatively alter the state or development of a system”^1^. In relation to Earth’s systems, such as the climate, a tipping point is a critical juncture at which the system responds nonlinearly to a small change in forcing, significantly altering future states^1,2^. A key climate tipping point has been projected to occur if global temperatures rise beyond 1.5 °C above pre-industrial levels, at which point threats to health, food security, water supply, economic growth, and geopolitical stability are expected to increase appreciably^3^.

In an ecological context, a tipping point is a condition in which an ecosystem experiences a shift to a new state, with significant changes to biodiversity, and the functions and services it underpins^4^. Several ecological tipping points have already been reached due to climate change and other anthropogenic influences, such as overfishing and pollution, resulting in significant loss of habitat, species, and biodiversity^5–8^, with a major decline in marine biodiversity since the mid-twentieth century^9^. However, individual components of Earth’s physical, ecological, chemical, and human systems will reach tipping points at different stages based on their internal dynamics and sensitivity to external forcing. For example, coral reefs are sensitive to ocean temperature, water quality, and pH, with recent, relatively moderate changes in ocean temperature and chemistry having resulted in significant degradation of this ecosystem^10^, possibly pushing reef sustainability beyond a tipping point^11,12^. Human populations on low-lying coral atolls, which depend on the reefs for protection from wave-driven flooding^13^, are also vulnerable, but could develop adaptation options that provide several more decades protection before sea level rise (SLR) reaches a critical threshold for their sustainability^14^. Defining tipping points for communities is, therefore, quite complex^15^. A consensus definition for what constitutes a community-scale tipping point has not been established^16–18^. How humans respond to physical climate disturbance is dependent on factors that include accommodation space, adaptability, migration potential, and habitat availability, many of which are controlled by anthropogenic factors, such as urbanization and land use change, as well as political will^19–25^.

SLR is one of the major consequences of climate change^26–28^ and is linked to global surface temperature^29^. Multiple studies derive SLR estimates whose mid-range values by 2100 exceed 1 m (e.g. Refs.^29–32^) with higher estimates (but with lower probabilities) exceeding 2 m under upper end emission and temperature scenarios^33,34^. During the Last Interglacial Stage (125,000 years ago), sea level was 6–9 m higher than present, serving as a potential analogue for the long-term (i.e. centuries to millennia) ramifications of present-day warming of 1–2 °C above pre-industrial levels^35^. With over 1 billion people expected to live in the coastal zone (< 10 m elevation) by 2050 (ref. ^36^), the potential permanent inundation and more frequent flooding of coastal environments and threats to human populations could have widespread and catastrophic impacts^37^.

The vulnerability of the coastal zone environment is often assessed by a single component deemed to be representative of that system, typically based on physical^38^, biological^11^, or statistical relationships^39^. Climate adaptation plans are generally developed with a single tipping point target, such as flood exposure. This may be an effective approach in identifying the vulnerability and timing of an individual component of the coastal zone (e.g., human population), but this approach inherently is not adequate to identify the full breadth of vulnerability of coastal systems to climate change (e.g. impacts to natural systems as well as built or human systems). Adaptation planning can benefit from effective identification of multiple SLR tipping points that will result in major and irreversible consequences to coastal ecosystems, such as sandy beaches and tidal wetlands, which provide valuable ecosystem functions and services (e.g. preservation of native biodiversity, nursery habitat and food chain support for rare and endangered biota, fish and wildlife, organic matter and nutrient cycling, coastal flood protection, cultural and recreational opportunities and associated economic revenue), as well as to coastal infrastructure and the human population. Moreover, better characterizing multiple tipping points, and their potential interactions, could help communities better understand the complexity of positive feedbacks^40^ and interconnected and cascading impacts resulting from social-natural dependence and interactions^41^.

Here we report on an interdisciplinary study to simultaneously assess the vulnerability of major components of a coastal system to climate change during the twenty-first century in the Santa Barbara region, California (USA). Our investigation integrates the modeled SLR response of beaches, cliffs, beach and tidal wetland ecosystems, flooding potential, and socioeconomic exposure to these flood hazards. Through this synthesis we demonstrate that multiple SLR tipping points exist, the identification of which is critical for effective short-and long-term planning, serving as a model for climate adaptation studies in coastal settings worldwide.

### Study approach

**Figure 1:**
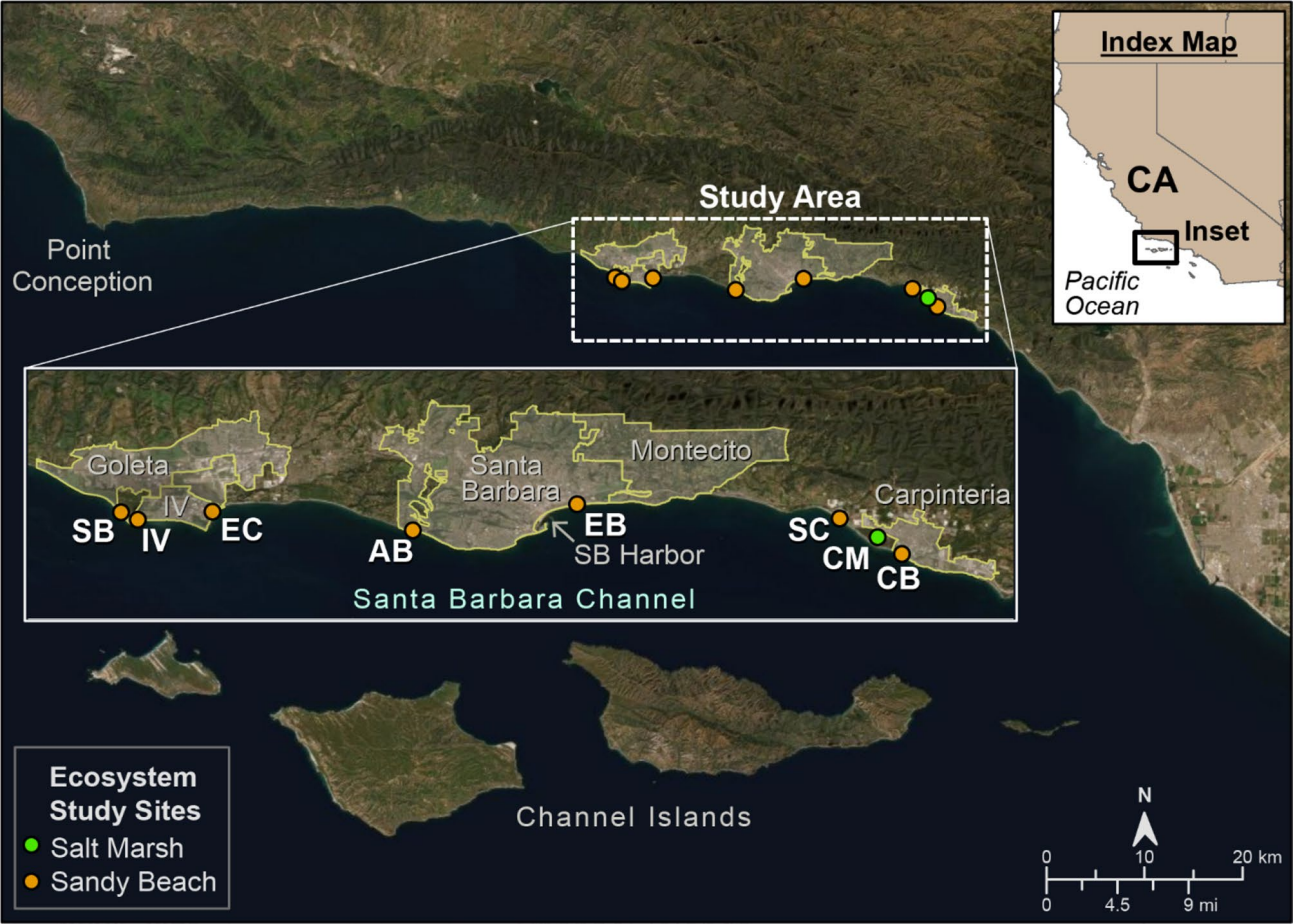
Study area. Projections of coastal change were conducted from Point Conception east to the Santa Barbara County line, just east of Carpinteria, California. Inset shows location of tidal marsh and sandy beach ecosystem study sites (SB = Sands/Ellwood beach, IV = Isla Vista, EC = East Campus, AB = Arroyo Burro, EB = East Beach, SC = Santa Claus, CM = Carpinteria salt marsh, and CB = Carpinteria city beach). Map also identifies the communities used to characterize socioeconomic exposure to projected flooding, including Goleta, Isla Vista, Santa Barbara (city), Montecito, and Carpinteria. (Figure generated using ArcGIS v. 10.4.2, www.esri.com. Local basemaps from https://services.arcgisonline.com/ArcGIS/rest/services/World_Imagery/MapServer).

We performed a multidisciplinary climate vulnerability study by integrating twenty-first century projected changes in climate forcing with the associated response of coastal watersheds, beaches, and tidal wetlands, and the exposure of the urbanized environment to flooding and erosion. A Mediterranean climate exists in the Santa Barbara region, characterized by warm, dry summers, and sporadically wet, cool winters^42^, with atmospheric rivers and quasi-periodic El Niño events strongly influencing local hydrology^43^ and coastal hazards^44,45^. This is an optimal location to perform such a study, as projected SLR in this region is substantial^30^ and it contains a representative swath of coastal environments along the 95-km study area (Fig. 1), including coastal bluffs and cliffs, dunes, beaches, river mouths, and tidal wetlands, with development ranging from rural to urbanized. Further, the influence of coastal hazards (e.g., erosion and flooding) on the ability of ecosystems to adapt is dependent on the variability and degree of urbanization.

The foundational study was designed to broadly support local government officials tasked with developing plans to adapt to climate-driven threats to natural and human communities^46^. Downscaled twenty-first century climate projections that included rainfall, winds, and SLR^47^ drove a coastal hazards model, the Coastal Storm Modeling System (CoSMoS)^48,49^, as well as an analysis of local impacts to watersheds^50^, tidal wetlands^46^, sandy beach ecosystems^51^, and socioeconomic exposure^52^. Each of the research components used a consistent set of atmospheric forcing and SLR scenarios provided by downscaled climate projections.

For this study, potential tipping points were defined by identifying the SLR scenarios at which subsequent projected changes to system metrics (e.g. ecosystem habitat-rich zones, amount of developed land in flood-hazard zones) exceed 50% compared to current conditions (i.e. zero SLR and no storm scenarios). We assume that a negative change of at least 50% of the total metric represents a substantial change in state at which point further degradation is likely^53,54^. Further, we assume that projected changes at a specific SLR increment cannot be reversed without human intervention, and therefore a potential tipping point has been reached. We also highlight SLR scenarios where the *relative* increase in projected change between two SLR scenarios exceeds 50%, less as a potential tipping point of system function and more as a moment at which the system experiences a considerable change that may mobilize communities to implement interventions to reduce future losses. We demonstrate that the timing of the projected changes for total system metrics and between SLR scenarios for the different components of this coastal system are out of phase, which can have implications for natural resource management and climate adaptation planning.

## Results

**Figure 2:**
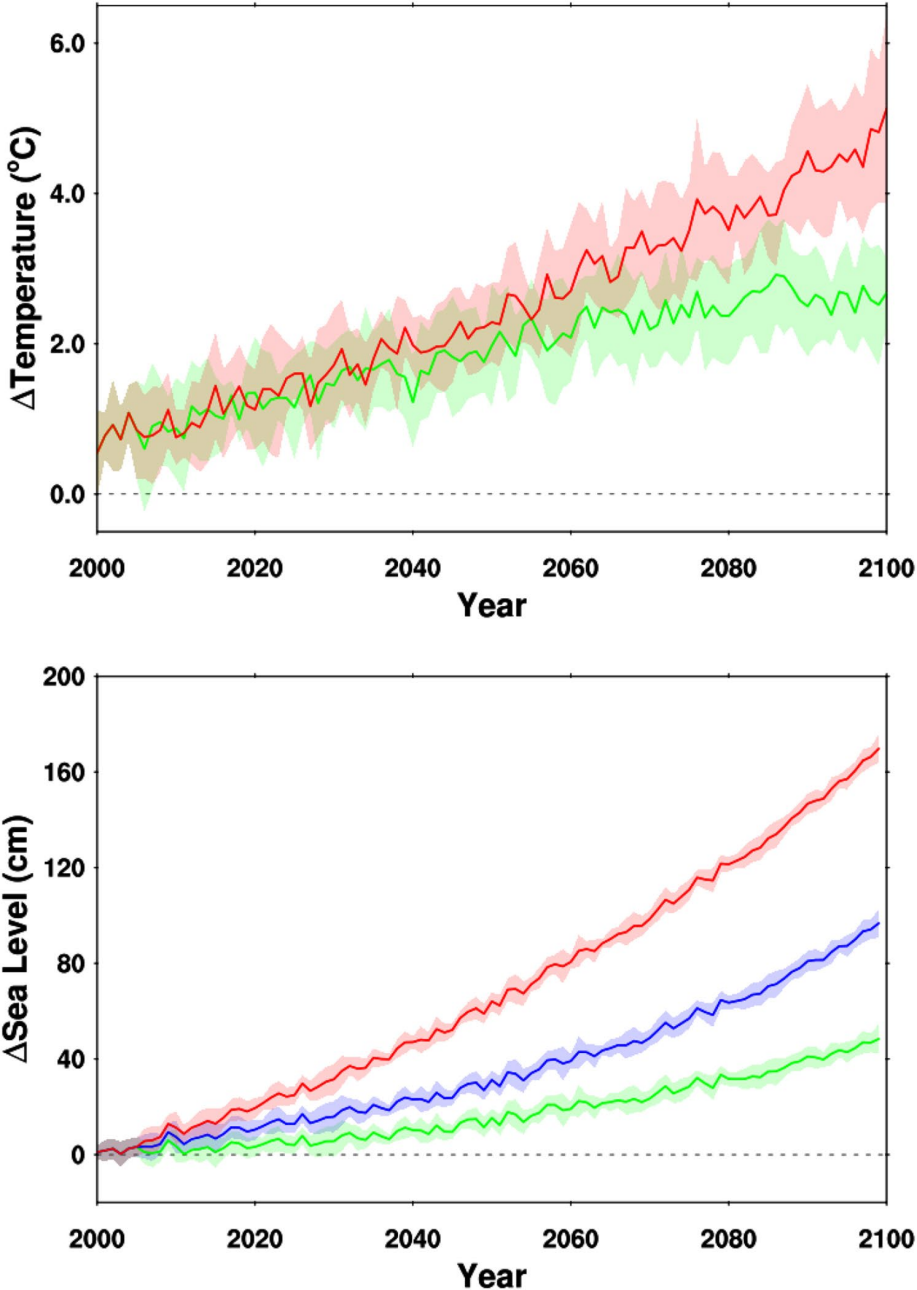
Global Climate Model projections of climate metrics for the Santa Barbara, California, region through 2100. Top panel: Annual mean temperature anomalies (relative to 1970–1999 base period) in the Goleta-Santa Barbara-Carpinteria coastal region from an ensemble of ten climate models employing the RCP 4.5 (green) and RCP 8.5 (red) emission scenarios. Solid line is the ensemble mean while the envelope is + /− one standard deviation of the individual model annual values from the ten model ensemble mean. Bottom panel: Departures of annual mean sea level (relative to year 2000 values) using three sea level rise scenarios and eight climate model projections using the RCP 8.5 emission scenario. The sea level rise scenarios are the low-range (green), mid-range (blue), and high-range (red) estimates from the National Research Council (NRC) 2012 report^85^. The solid line represents the ensemble mean while the envelope is + /− one standard deviation of the annual values from the eight models about the ensemble mean.

The rate of twenty-first century SLR is dependent on surface temperature increases^29^, which in turn are dictated by prescribed global emissions trajectories, characterized by the Representative Concentration Pathway (RCP) scenarios developed for the 5th Assessment Report of the Intergovernmental Panel on Climate Change^55^. For the Santa Barbara region, two RCP scenarios were considered: the intermediate RCP4.5 scenarios wherein emissions peak in mid-21st Century and diminish thereafter; and the higher RCP8.5 scenario wherein emissions continue to rise along with population growth through the 21st Century. Projected global surface temperatures for the 21st Century were downscaled for both RCPs to the study region using 10 global climate models (GCMs) (Fig. 2). Using the 30 year 1970–1999 average as a recent baseline, the two RCP scenarios follow a similar temperature trend until 2035, increasing by ~ 1.5 °C. After 2035 the two scenarios begin to diverge, and by the end of the 21st Century have increased by ~ 2.5 °C under RCP4.5 and ~ 5.0 °C under the RCP8.5 scenario. There has been a marked rising trend of recent global surface temperatures, which in 2019 has already reached ~ 0.55 °C above the 1970–1999 baseline, and ~ 1.2 °C above pre-Industrial temperatures^56^. Moreover, the years 2015–2020 were the 6 warmest in the 140-year instrumental record^56–58^. Thus, by 2029 the Paris Agreement target of 1.5 °C will likely be reached under either RCP4.5 or 8.5, and possibly sooner^59^, which is widely considered a critical tipping point for climate impacts to natural and human systems^3^.

As recent temperatures are rising at a rate equal or higher than either RCP4.5 or RCP8.5 scenarios, it seems prudent to consider future SLR that accords with the higher RCP8.5 scenario. Downscaled wind, surface pressure and temperature from eight RCP8.5 GCMs were employed to drive short period sea level variability, superimposed upon three long term SLR scenarios (Fig. 2). These scenarios are consistent with recent SLR guidance from the State of California^30,32^. Mid-century SLR projections (2050) for the Santa Barbara region range from a median of 15 cm (low range) to 60 cm (high range), and 45 cm to 170 cm by 2100. An extreme scenario that considers rapid warming coupled with accelerated ice sheet decay projects as much as ~ 3 m of SLR by the end of the century for Santa Barbara^31,32,47^.

**Figure 3:**
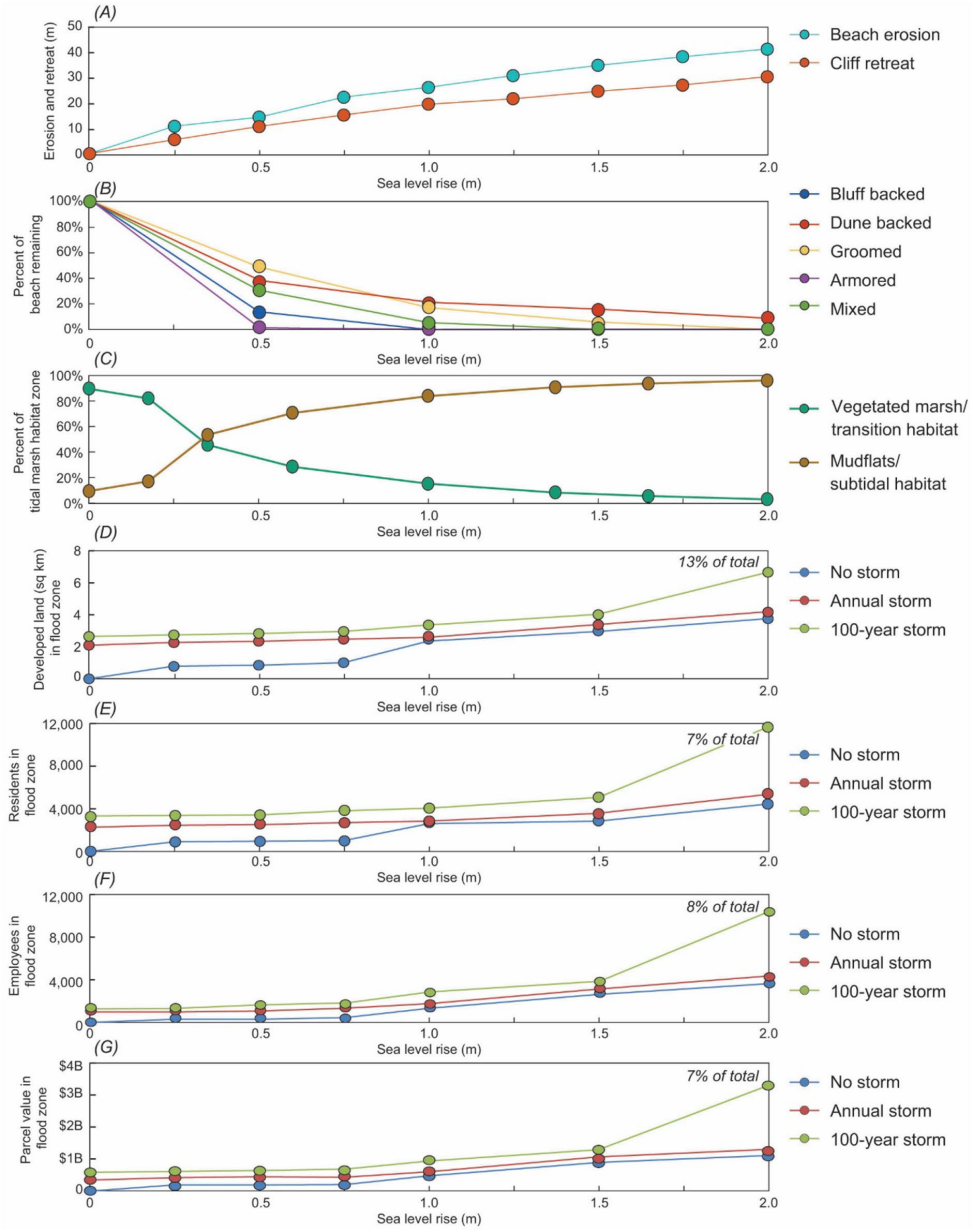
Projected changes in coastal systems for the Santa Barbara study area. (**A**) Beach erosion and cliff retreat projections, (**B**) the percent of beach remaining along transects, (**C**) the percentage of vegetated marsh/transition habitat (upland, transition zone, and high/mid marsh) and high/low mudflat and subtidal habitat in Carpinteria salt marsh, and the amount of various community assets in projected flood zones including (**D**) developed land, (**E**) residents, (**F**) employees, and (**G**) parcel values relative to sea level rise scenarios from 0 to 2 m.

Beaches and cliffs provide the first lines of defense for SLR and storm-driven hazards that can result in the more frequent exposure and degradation of in situ and adjacent ecological and human systems. For the Santa Barbara study area, the relative amounts of beach erosion and cliff retreat are projected to increase most significantly beyond 0.50 m (55%) and 0.25 m (95%) of SLR, respectively (Fig. 3A). Coastal erosion rates would be expected to accelerate as the rate of SLR increases under the higher scenarios, but this is not projected for the Santa Barbara region as coastal defenses and other urban infrastructure limit landward retreat in many locations.

The future width of the ecologically important upper intertidal zone of sandy beaches in the Santa Barbara region, whose landward boundaries included coastal bluffs, dunes, and armoring as well as intensively groomed beaches, was assessed for SLR scenarios ranging from 0 to 2.0 m in 0.5 m increments (Fig. 3B). Using CoSMoS projections of the mean high water (MHW) shoreline and maximum water levels along the beaches, projected future upper intertidal zone habitat sharply decreased immediately with the transition from 0 to 0.5 m SLR, averaging 74% habitat loss across all beach types, including 87% and 99% for the bluff-backed and armored beaches, respectively, that represent the majority of beaches in the study region, with a resulting loss of ecosystem functions and services^46^. When SLR reaches 1.0 m, < 10% of the upper tidal zone habitat space remains, and when SLR reaches 1.5 m SLR, < 5% remains. Analyzing the loss of the upper intertidal zone when considering SLR combined with annual storms yielded identical tipping point patterns (Supplemental Table S1). With the most severe ecosystem habitat loss observed after the first SLR scenario (i.e., between 0 and 0.5 m), these results indicate that a tipping point for sandy beach ecosystems may have already been reached.

Tidal marsh habitat zones in Carpinteria salt marsh were tracked for SLR scenarios based on the present-day relationship between habitat distribution, elevation and flooding frequency (Fig. 3C). Although mid-marsh, high marsh, and transition-upland zones currently account for > 90% of the tidal marsh habitat areas, with just 0.36 m of SLR relative to the marsh surface, those zones are projected to comprise only 46% of the tidal marsh, with the consequent loss of ecological function, including nursery and foraging habitat and biodiversity of rare, threatened and endangered species^46,60–62^. With increasing SLR, the mudflats and sub-tidal habitats become dominant, including a more than tripling of the lower mud flat area when transitioning from 0.18 to 0.36 m of SLR, and the high mudflat zone between 0.36 and 0.61 m of SLR. During the SLR transition from 0.18 to 0.61 m, areas of the mid-marsh, high marsh, transition, and upland zones decrease most acutely by 54%. Upon reaching 1 m of SLR, mud flats and sub-tidal regions are projected to account for 85% of the tidal marsh, and 97% at 2 m. In short, by ~ 0.6 m of SLR, consistent with sandy beach ecosystems by 0.5 m of SLR, vegetated tidal marsh habitat will have already severely degraded, with the largest change occurring after 0.18 m of SLR. Since the habitat changes are relative to the marsh surface, the timing of the tipping point depends on the rate of accretion and SLR scenario. Assuming a marsh accretion rate of 4 mm/year^63^ and the high range scenario of SLR, mudflat would comprise 56% of habitat by 2050 and > 80% by the end of the twenty-first century^46^, representing a major shift in habitat type.

The potential for socioeconomic tipping points was based on projected changes in the number of residents and employees and the amount of developed land and parcel values in areas that are estimated to have daily/permanent coastal flooding (i.e. inundation) and storm-driven flooding (i.e. annual and 100-year return interval events) relative to CoSMoS SLR scenarios (0 to 2.0 m). Changes in flood-hazard exposure were estimated for five communities near the sandy beach and tidal wetland ecosystem study sites, including Goleta, Isla Vista, Santa Barbara, Montecito, and Carpinteria (Fig. 1). Exposure to daily flooding (i.e. no storms considered) for each societal metric relative to study area totals is far below the 50% tipping point threshold, instead only directly affecting 2.4% of total parcel values, 2.7% of total residents, 2.9% of total employees, and 7.4% of total developed land even for the largest SLR scenario of 2.0 m (Fig. 3D–G). Although total system exposure is not projected to surpass the 50% threshold observed in the ecological systems, there are certain SLR scenarios that could result in considerable, relative increases between SLR scenarios that may galvanize communities to mitigate further losses. For example, the largest relative increases of societal exposure to inundation occurs across all four metrics when transitioning from 0.75 m to 1.0 m of SLR (Fig. 3D–G, Supplemental Table S1), with an increase of 1,712 residents (175% increase), 972 employees (254% increase), $283 million dollars of parcel value (160%) and 1.5 km^2^ of developed land (144% increase). Peak increases in coastal flood hazard exposure associated with SLR inundation are preceded by the largest increases in the relative amounts of beach erosion and cliff retreat (Fig. 3A).

Even without considering future SLR, the Santa Barbara region is already at risk from coastal storm, wave-driven flooding hazards (Fig. 3D–G). The five communities in our study area collectively have 2,302 residents and $345 M in property value in projected flood areas for coastal storms with an average annual return interval, increasing to 3,232 residents and $581 M in property values for storms with a 100-year return interval. These storm-related exposure values rise considerably when taking into account the role of SLR in increasing potential flood areas. Nevertheless, similar to the non-storm scenarios, total system exposure is not expected to surpass the 50% threshold for any societal metric, instead only directly affecting 3–7% of residents and parcel values, 3–8% of employees, and 8–13% of developed land for the largest SLR scenario of 2.0 m (range noting annual and 100-year storm estimates). There are, however, considerable relative increases in flood hazard exposure from 100-year storms when transitioning from 1.5 to 2.0 m of SLR (Fig. 3D–G, Supplemental Table S1), with an increase of 6,542 residents (130% increase), 6,532 employees (171% increase), $2B of parcel value (156%) and 2.6 km^2^ of developed land (66% increase). This analysis of the physical and societal hazards related to SLR demonstrates that the largest changes in exposure for the different styles of projected future flooding are asynchronous (e.g., when transitioning from 0.75–1.0 m of SLR for daily flooding/inundation compared to 1.5–2.0 m of SLR for storm-driven flooding). Further, when exposure changes are calculated uniformly by comparing scenarios based on jurisdictional and habit area totals and not relative changes, the human system values do not occur until much higher SLR scenarios, are far smaller, and do not qualify as tipping points.

## Discussion

The results described herein indicate that degradation of coastal ecosystems in the Santa Barbara region is on the leading edge of climate impacts, consistent with observations world-wide^5–8,10–12^. Sandy beach ecosystems have possibly reached a tipping point, and tidal marshes will reach that point perhaps as early as ~ 0.25 m of SLR. This timing (~ mid-century or sooner) is similar to the temperature tipping point of the terrestrial biosphere projected to be within just a few decades^64^, although the precise timing of this transition is difficult to determine due to uncertainty related to future climate warming, response of SLR to warming, SLR scenario resolution, and future sediment supply to beaches and marshes. Greenhouse gas emissions are currently tracking the RCP8.5 scenario most closely^59^, so warming is likely to rapidly exceed 1.5 °C and therefore SLR could reach 0.25 m by mid-century under either a middle or high SLR scenario, and just a few decades later for the low SLR scenario (Fig. 2). Even if greenhouse gas emissions are reduced to a net zero level, the current concentration of C0_2_ in the atmosphere has already committed oceans to an additional ~ 1.7 m of global mean SLR^65^, which would match the higher end SLR scenario projected for Santa Barbara by 2100. Given that 250 million people currently live within 1 m of present-day high tide across the world^66^, a SLR tipping point may have already been reached globally and locally.

Sandy beach ecosystems are especially vulnerable and appear on the verge or have already exceeded a tipping point, especially for armored beaches (e.g. refs. 67,68), where 99% of the existing upper beach zone habitat is projected to be lost with just 0.5 m of SLR (range of all beach types = 51–99%, mean = 74%). The majority of beaches are projected to decline in overall width with increasing SLR. Importantly, the loss of beach width will not be evenly distributed across intertidal zones. Upper beach zones are projected to experience the greatest declines in width and losses with SLR. Although often narrow in width, these upper intertidal zones are vital components of biodiversity and ecosystem function^51,69^. The sandy upper intertidal zone is associated with the distributions of key beach organisms, biodiversity and ecosystem functions, including the accumulation of macrophyte wrack and the wrack-associated invertebrate community^67,69^. This often narrow zone supports ~ 45% of total intertidal invertebrate biodiversity, provides prey resources for birds and fish, and plays a vital role in detrital processing and nutrient cycling^51,68–73^. These key upper beach zones are already scarce and/or ephemeral for many beaches in the study region with consequent loss of biodiversity and ecosystem function (e.g. refs. ^67,74,75^). When seawater regularly reaches the bluff toe, armoring structure or beach limit, drift macrophyte wrack and the rich intertidal biodiversity, ecosystem functions and prey resources that macrophyte supports, as well as critical habitat for fish and wildlife are eliminated from the beach ecosystem. The upper intertidal transition, the zone where native coastal strand vegetation develops that can enhance resilience by trapping and storing sand and building dune topography^76^, also is eliminated.

While continued cliff retreat might create more space for sandy beach habitats, SLR-driven erosion will remove the sand in front of cliffs. Across Southern California, one- to two-thirds of beaches, and the habitats therein, are expected be drowned and lost due to SLR this century, the loss accelerated by curbs on landward migration due to cliffs and/or urban resistance^77^. Existing coastal armoring already restricts the migration potential of up to 57% of Southern California county beaches, including 12% in Santa Barbara County^78^, and impacts intertidal biodiversity and function^67,68^. There has been a greater than five-fold increase in armoring over the last 50 years across this region, and as the coastal hazards associated with rising seas increase in this urbanized setting, as in others worldwide, more structures are likely to be constructed that protect human populations but limit habitat migration.

**Figure 4:**
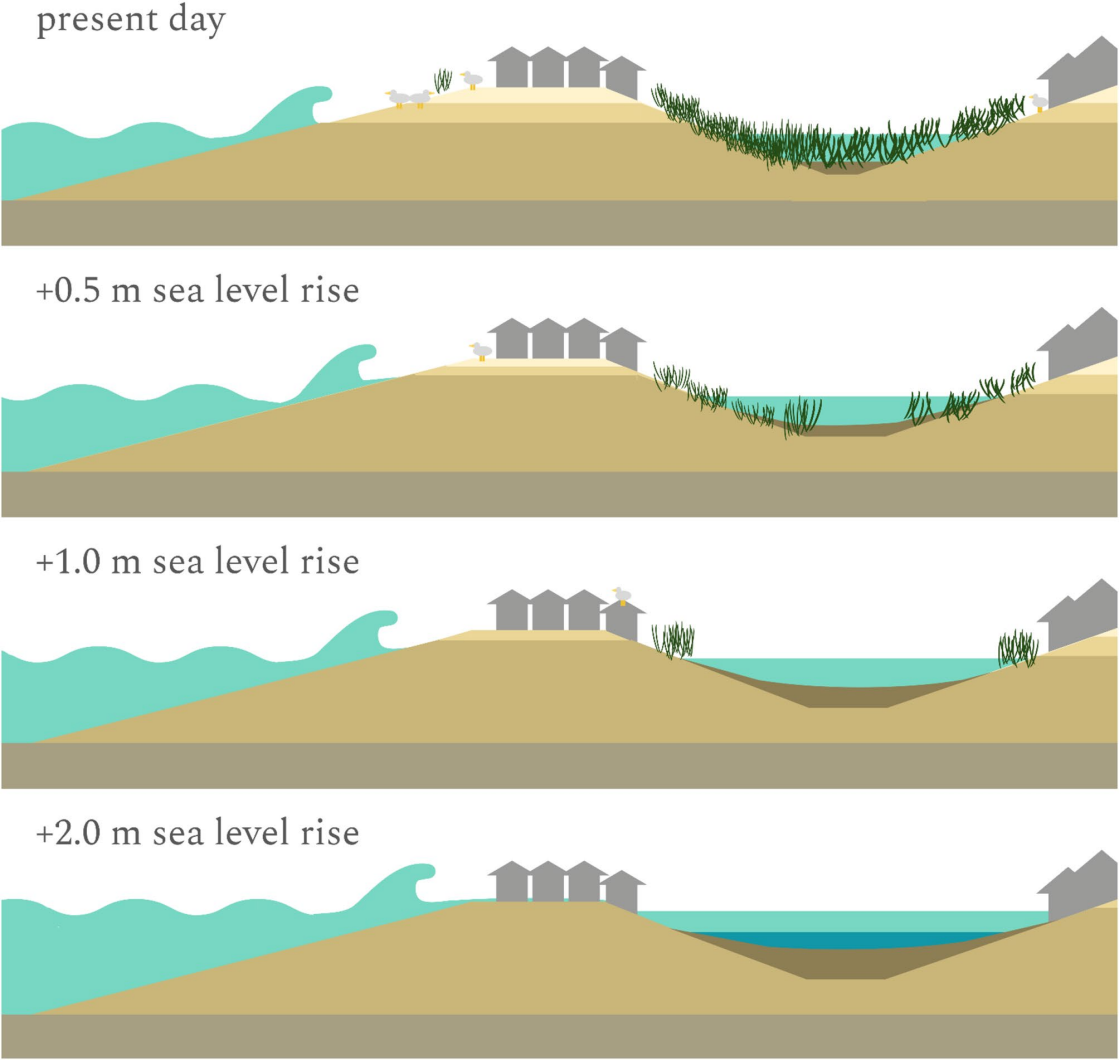
Conceptual diagram of the tipping points in the Santa Barbara coastal system with increasing sea level rise. Present day: coastal habitats and infrastructure vulnerable. 0.5 m sea level rise (SLR): sandy beach ecosystems squeezed by SLR and urban infrastructure, tidal marsh habitats degrading. 1 m SLR: sandy beach ecosystems and salt marsh habitats almost completely eliminated, daily tidal flooding impacts urban environments. 2 m SLR: Habitats lost, and urban environment highly susceptible to daily and periodic storm impacts.

Urbanization is a major factor in the observed habitat squeeze of sandy beach and tidal marsh ecosystems and related short-term tipping points as engineered tidal marsh shorelines, armored beaches, as well as bluffs prevent the upland transgression of tidal marshes and landward migration of sandy beaches (Fig. 4). In the absence of urbanization, these coastal habitats are resilient and capable of responding to high rates of SLR, as evidenced by their ability to survive during the > 100 m of SLR of the late Quaternary, including several centuries during Meltwater Pulse 1A where rates approximated 5 cm/year^79^.

However, tidal marshes have more recently been in decline across the coast of California due to urbanization and land management practices, with a 48% loss of habitat across southern California since 1850, including a 62% decline in Santa Barbara County^80^. Accelerating sea levels world-wide and limits to landward mobility will further increase tidal inundation of marshes leading to changes in key physical and biological properties known to structure marsh plant communities and habitats^81^. As demonstrated in this study, major changes are forthcoming or already underway in Carpinteria salt marsh, and this regionally scarce ecosystem may pass a tipping point with less than 0.25 m of SLR with the significant loss of high salt-marsh and transition habitats and the functions these habitats provide. Similar losses of existing vegetated marsh by the end of the century have been projected for other tidal marshes region-wide^82^ and are consistent with global studies linking early warning signals to marsh collapse^83^. The precise timing of habitat evolution will depend on the rate of SLR and the accretion rate of the marsh surface, but ultimately, with limited landward accommodation space, this vegetated marsh will be converted to mudflat. While specific tipping point dates are tidal marsh-specific and depend on local hydrology, sediment supply, and topography, urbanized tidal marshes are the norm along the California coast and other populated settings. This study, therefore, may serve as a proxy for the response of similar tidal marsh systems world-wide. Mitigating and adapting to the anticipated vulnerability of this ecosystem to climate-related impacts are a key planning and management priority for local, state, and federal agencies^84–86^.

If the sole trigger for communities across the region to implement adaptation options is based on substantial increases in the physical exposure of developed land and built systems to SLR and storms, then communities might delay adaptation strategies until after 2050 as the largest absolute and relative changes in exposure are not projected until SLR exceeds 0.75 m for daily flooding (i.e. inundation) and 1.50 m for storm-driven flooding, and therefore not projected to occur until toward the end of the twenty-first century or later. Further, when looking at socioeconomic exposure, the population, employees, property values, and developed land directly exposed to flooding in the study area jurisdictions represents just 2–13% of totals even under the most extreme SLR scenario considered here (i.e. 2 m), and the largest changes between scenarios are all 5% or less. However, societal exposure to other coastal hazards, such as tsunamis^87^ and groundwater hazards^88^, will also increase due to SLR but were not considered here.

Although projected changes in flood-hazard exposure may not represent high percentages of the total number or amount of residents, employees, property values and developed land in the communities, these values do not consider the interconnection of the human-natural system. For instance, the study region is highly dependent on tourism, driven largely by visitors enjoying the beaches and wetlands along the coast. For example, an analysis of visitor profiles in the study area identified several activities that relate to these coastal systems, including going to the beach (52–68% of visitors, depending on type of visitor), going to parks (19–25%), and water-based recreational activities (2–8%). This report also estimates that the region had an average of 28,884 daily visitors, which supported 13,482 jobs and resulted in $1.9 billion in annual revenue^89^. While there is not a direct 1:1 relationship between beach loss and loss of tourism, it has been shown to lead to economic impacts in other regions and is something for which coastal communities could prepare. In addition, valuable public infrastructure and public services, such as the Santa Barbara airport, the railroad, Highway 101, the El Estero Water Resource Center, along with stormwater drains, sewage pump stations, and harbors are located in these coastal strips; even intermittent coastal storm-related flooding can cause severe interruptions of these systems, resulting in further economic impacts that can compound over time. Understanding the timing of different tipping points (or at least the point when the largest changes in exposure are projected to occur), including losses to natural systems, upon which economic drivers such as coastal tourism are dependent, could therefore be important for robust adaptation planning^15^.

Admittedly, assuming an irreversible change in our tipping point definition does not consider the adaptive capacity of each system to respond to SLR. Adaptive capacity may be limited when beach and tidal marsh habitat areas are squeezed between rising seas and hardened urban landscapes. In such cases, endemic plant and animal populations dependent on those habitats may be completely eliminated. Other biota (e.g. deeper water fish assemblages) might use the flooded habitat, however, overall ecological composition, biodiversity, and functions will change. Conversely, while the socioeconomic impacts of more frequent coastal flooding may be severe, human populations have the ability to migrate to safer, inland settings, although social inequities can play a major role in migration potential for underserved communities. The complexity of adaptive capacity, human response, and societal value placed on different coastal systems makes a universal definition for a community-scale tipping point difficult to quantify in a way that directly supports management action across the board. What is clear, however, is that regardless of the chosen metric, individual coastal systems will continue to evolve at different rates and reach critical thresholds at different times due to climate change, prompting the need to adopt a multi-tiered, multi-disciplinary approach to address the range of physical, biological, and human impacts associated with climate change in coastal systems.

The most substantial changes and/or tipping points identified here are based on projections of future coastal hazard exposure and ecosystem response assuming no interventions, i.e., they are not inevitable. Measures that could counter local SLR and storm impacts include ecosystem restoration, removing barriers to inland transgression^90^, increasing sediment supply (e.g. dam removal), and removing shoreline armoring and reducing mechanized beach grooming^76^. Ultimately, the pace of management action will depend upon societal values and the availability of community resources (e.g. financial resources and political will) to mitigate potential losses, absorb losses, and/or implement adaptation measures within the coastal zone. However, if communities only look at singular triggers to make adaptation planning decisions – either for natural (e.g. limited marsh conversion) or human systems (e.g. number of businesses impacted) – they are missing the interconnectedness of natural-human systems. The full range of vulnerability, and their associated tipping points, must be analyzed in tandem. If the prevention of significant ecosystem changes is a priority, then swift action is essential as those tipping points are imminent. Similarly, beyond the intrinsic value of having thriving coastal ecosystems, like many coastal towns, a healthy coastal zone is also critical to this region’s economy.

In summary, defining a tipping point that universally captures the point of significant degradation across physical, biological, and human systems is challenging. Cascading interactions between SLR, coastal hazards, and human response amplify impacts but are complex and difficult to quantify; humans could choose to adapt and stay in place or move away from a hazardous region. Moreover, in a tourism-driven economy such as Santa Barbara’s, focusing only on flooding to infrastructure and property, and not preparing for the impacts to the coastal ecology and beach loss, ignores an important socio-natural interconnection on which the region’s economy depends; natural system degradation will likely negatively impact coastal tourism, which in turn then makes local businesses and communities vulnerable well before direct flood exposure to physical assets. Nevertheless, to better inform resource decisions, this study identifies climate-adaptation planning that considers tipping points for multiple components of a coastal system, including for natural ecosystems, as opposed to the more common singular focus on human components.

## Materials and methods

### Climate modeling

Ten global climate models (GCMs) were selected from the Coupled Model Intercomparison Project Phase 5 (CMIP5) archive^55,91^ based on their realism in representing California’s historical climate^47^. The Local Constructed Analogs (LOCA) statistical technique was used to downscale each GCM to 6 km resolution for daily temperature and precipitation from 1950–2100 for Representative Concentration Pathway (RCP) scenarios RCP4.5 and RCP8.5 (ref. ^92^).

Sea level rise projections for the 21st Century were derived from the National Research Council^85^ and integrated with short period fluctuations due to tides, meteorological conditions, and short period climate variability (e.g. El Niño) to produce hourly coastal water levels for the study area. Model inputs for the multiple-linear regression model were based on water level observations at Santa Barbara Harbor and historical NCEP meteorological reanalysis data, with the variables including daily climate model data, surface pressure, wind stress, and both local sea surface temperature and central Pacific Ocean sea surface temperature to assess El Niño variability^93^.

### Coastal hazards

Driven by the wind and pressure fields from the native resolution and downscaled GFDL-ESM2M (RCP4.5 scenario) GCM from above, the Coastal Storm Modeling System (CoSMoS)^48,49,94,95^ was applied to the study area by considering 40 SLR (i.e. 0, 0.25, 0.50, 0.75, 1.00, 1.25, 1.50, 1.75, 2.00 and 5.00 m) and storm scenarios (i.e. daily conditions and annual, 20-year and 100-year storms). Twenty-first century wind fields were fed into a global and nested Eastern North Pacific WAVEWATCH III wave model^96^ to establish wave conditions at the continental shelf edge^97^, and then dynamically downscaled using SWAN^98^ to transform the waves to the nearshore. To establish nearshore water level boundary conditions, SWAN was coupled with DELFT3D-FLOW to capture the tides, seasonal water level anomalies, river discharge and storm surge^95,99,100^. High-resolution hydrodynamic grids (O ~ 10 m) were used to model total water levels and overland flooding for complex shorelines, including protected embayments, while cross-shore, 1-D XBeach models^101^ were spaced every 100 m alongshore on the open coast to predict wave set-up and swash. Projected flood levels for each scenario were interpolated onto a 2-m grid and differenced from a Digital Elevation Model (DEM)^102^ to provide the flood extent and depth. Storm scenarios for the full computational application of CoSMoS were established via a proxy approach^103^, which also served to provide a twenty-first century, continuous time series of total water levels to drive the coastal change models for sandy beaches (CoSMoS-COAST)^77^ and cliffs^104^ along the established XBeach transects. The long-term coastal change projections were used to evolve the DEM for the future flooding scenarios^105^. All the model projections are freely available for viewing^106^ and download^107^.

### Sandy beach ecosystems

Standard elevational metrics were related with ecological components and habitat zones of beaches to identify the vulnerability of sandy beach ecosystems to SLR. The current and future state of the ecologically critical, upper tidal zone of each beach, which ranged from bluff-backed, dune-backed, armored, and groomed, was measured and modeled using the total water level datum^108,109^ as a proxy for the dynamic landward extent of the upper intertidal zone, equivalent to the daily High Strand line (HTS)^51^. Ecological research on area beaches^51,67,69–71^ combined with beach surveys and coastal processes studies enabled a predictive framework to be established between SLR and changes in the upper beach zones^84,110^. The total water level projections from CoSMoS^48,49,95^ for 0.50, 1.00, 1.50, 2.00 and 5.00 m of SLR combined with background conditions (i.e. daily) and annual storm scenarios were used to establish the landward extent of the upper beach zone. The seaward limit of the upper beach zone was determined by the projected MHW elevation, as determined by shoreline modeling driven by a CoSMoS-generated twenty-first century water level time series^77^. Upper beach zone landward migration and width was restricted by the presence of non-erodible structures, such as revetments, sea walls, roads or parking lots.

### Tidal wetlands

This study examined the wetland evolution of Carpinteria salt marsh, a 93 ha tidal wetland (Fig. 1). This wetland represents an urbanized marsh system, surrounded by urban and residential development that restricts potential upland migration, and an engineered inlet that maintains the tidal connection to the ocean. The system contains a wide range of marsh species and habitats, including salt tolerant pickleweed (*Salicornia pacifica*) dominating the regularly flooded middle tidal marsh, and various succulent, grass, and perennial and annual herb species that occur in the high marsh and upland transition zones, including rare and endangered species^46,62^. Existing habitats were delineated using multispectral aerial imagery as open water subtidal, high and low mudflat, coastal salt marsh, (mid and high), transition, and undeveloped upland. Using Santa Barbara Harbor tide data from 2006–2014 (ref. ^111^) and elevation surveys from both aerial Lidar and in situ Real Time Kinematic Global Positioning System (RTK GPS), habitat was linked to elevation and flooding frequency^46,62^. This relationship was then applied to assess future marsh habitat evolution using SLR ranging from 0 to 2.5 m. The potential timing of habitat evolution was evaluated assuming a vertical accretion rate of 4 mm/year^63^ and based on the National Research Council SLR scenarios^85^.

### Socioeconomic exposure

Socioeconomic exposure to the flood extent for 21 SLR/storm scenarios^49^ was estimated by the geospatial analysis of various community assets^52,112^. Residential counts are based on 2010 block level data from the U.S. Census Bureau^113^. Employee counts and locations came from the 2020 Infogroup Employer Database^114^. Parcel boundaries and their total assessed values (tax year 2019) are from the Homeland Infrastructure Foundation-Level Data (HIFLD) repository^115^. Estimates of developed land are based on high-, medium-, and low-intensity developed classes in the 2016 National Land Cover Database^116^. All the data are served up in an interactive web application^117^.

## Supplementary Information

Supplementary Information.

## References

[1] Lenton TM (2008). Tipping elements in the Earth’s climate system. Proc. Natl Acad. Sci. USA.

[2] Lenton TM (2011). Early warning of climate tipping points. Nat. Clim. Chang..

[3] Intergovernmental Panel on Climate Change, 2018: Global Warming of 1.5°C. An IPCC Special Report on the impacts of global warming of 1.5°C above pre-industrial levels and related global greenhouse gas emission pathways, in the context of strengthening the global response to the threat of climate change, sustainable development, and efforts to eradicate poverty. Masson-Delmotte, V. *et al.*, Eds. (2018).

[4] Secretariat of the Convention on Biological Diversity, Global Biodiversity Outlook (GBO) 4 (Convention on Biological Diversity, Montréal, 2014), 155 pp. (2014).

[5] Cox PM, Betts RA, Jones CD, Spall SA, Totterdell IJ (2000). Acceleration of global warming due to carbon-cycle feedbacks in a coupled climate model. Nature.

[6] Hughes L (2000). Biological consequences of global warming: is the signal already apparent?. Trends Ecol. Evol..

[7] McCarty JP (2001). Ecological consequences of recent climate change. Conserv. Biol..

[8] Mantyka-Pringle CS, Martin TG, Rhodes JR (2012). Interactions between climate and habitat loss effects on biodiversity: a systematic review and meto-analysis. Glob. Chang. Biol..

[9] Duarte CM (2020). Rebuilding marine life. Nature.

[10] Hoegh-Guldberg O (2007). Coral reefs under rapid climate change and ocean acidification. Science.

[11] Mumby PJ, Hastings A, Edwards HJ (2007). Thresholds and the resilience of Caribbean coral reefs. Nature.

[12] Mora C, Graham NAJ, Nyström M (2016). Ecological limitations to the resilience of coral reefs. Coral Reefs.

[13] Reguero BG (2021). The value of US coral reefs in flood risk reduction. Nat. Sustain..

[14] Storlazzi CD (2018). Most atolls will be uninhabitable by the mid-21^st^ century because of sea-level rise exacerbating wave-driven flooding. Sci. Adv..

[15] van Ginkel KCH (2020). Climate change induced socio-economic tipping points: review and stakeholder consultation for policy relevant research. Environ. Res. Lett..

[16] Andersen T, Carstensen J, Hernandez-Garcia E, Duarte CM (2009). Ecological thresholds and regime shifts: approaches to identification. Trends Ecol. Evol..

[17] Scheffer M (2009). Early-warning signals for critical transitions. Nature.

[18] Petraitis PS, Hoffman C (2010). Multiple stable states and relationship between thresholds in processes and states. Mar. Ecol. Prog. Ser..

[19] Richards JA, Mokrech M, Berry PM, Nichols RJ (2008). Regional assessment of climate change impacts on coastal and fluvial ecosystems and the scope for adaptation. Clim. Change.

[20] Vos CC (2008). Adapting landscapes to climate change: examples of climate-proof ecosystem networks and priority adaptation zones. J. Appl. Ecol..

[21] Kwadijk JCJ (2010). Using adaptation tipping points to prepare for climate change and sea level rise: a case study in the Netherlands. Wiley Interdiscip. Rev. Climate Change.

[22] Haasnoot M, Kwakkel JH, Walker WE, ter Maat J (2013). Dynamic adaptive policy pathways: a method for crafting robust decisions for a deeply uncertain world. Global Environ. Change.

[23] Haasnoot M (2020). Adaptation to uncertain sea level-rise; how uncertainty in Antarctic mass-loss impacts the coastal adaptation strategy of the Netherlands. Environ. Res. Lett..

[24] Torio DD, Chmura GL (2013). Assessing coastal squeeze of tidal wetlands. J. Coastal Res..

[25] Bragg WK, Gonzalez ST, Rabearisoa A, Stoltz AD (2021). Communicating managed retreat in California. Water.

[26] Intergovernmental Panel on Climate Change. Climate Change 2007: The Physical Science Basis. Contribution of Working Group I to the Fourth Assessment Report of the Intergovernmental Panel on Climate Change, Solomon, S. *et al.*, Eds. (Cambridge Univ. Press, Cambridge) (2007).

[27] Nichols RJ, Cazenave A (2010). Sea-level rise and its impact on coastal zones. Science.

[28] Hinkel J (2014). Coastal flood damage and adaptation costs under 21st century sea-level rise. Proc. Natl. Acad. Sci..

[29] Rahmstorf S (2007). A semi-empirical approach to projecting future sea-level rise. Science.

[30] Griggs, G. B. *et al.* Rising seas in California: an update on sea-level rise science. California Ocean Science Trust, 71 pp (2017).

[31] Sweet, W. V. *et al*. Global and regional sea level rise scenarios for the United States. NOAA Technical Report NOS CO-OPS 083, NOAA/NOS Center for Operational Oceanographic Products and Services (2017).

[32] Ocean Protection Council, State of California sea level rise guidance, 2018 update. California Natural Resources Agency, 84 pp. http://www.opc.ca.gov/webmaster/ftp/pdf/agenda_items/20180314/Item3_Exhibit-A_OPC_SLR_Guidance-rd3.pdf (2018).

[33] Le Bars D, Drijfhout S, de Vries H (2017). A high-end sea level rise probabilistic projection including rapid Antarctic ice sheet mass loss. Environ. Res. Lett..

[34] Bamber JL, Oppenheimer M, Kopp RE, Aspinall WP, Cooke RM (2019). Ice sheet contributions to future sea-level rise from structured judgement. Proc. Natl. Acad. Sci..

[35] Kopp RE, Simons FJ, Mitrovica JX, Maloof AC, Oppenheimer M (2009). Probabilistic assessment of sea level during the last interglacial stage. Nature.

[36] Merkens J-L, Reimann L, Hinkel J, Vafeidis AT (2016). Gridded population projections for the coastal zone under the Shared Socioeconomic Pathways. Global Planet. Change.

[37] Wong, P. P. *et al.*, Coastal systems and low-lying areas. In *Climate Change 2014: Impacts, Adaptation, and Vulnerability. Part A: Global and Sectoral Aspects. Contribution of Working Group II to the Fifth Assessment Report of the Intergovernmental Panel on Climate Change, Field*, C.B. *et al.*, Eds., Cambridge University Press, pp. 361–409 (2014).

[38] Turner RE, Kearney MS, Parkinson RW (2018). Sea-level rise tipping point of delta survival. J. Coast. Res..

[39] Sweet WV, Park J (2014). From the extreme to the mean: acceleration and tipping points of coastal inundation from sea level rise. Earth’s Future.

[40] Kopp RE, Shwom RL, Wagner G, Yuan J (2016). Tipping elements and climate-economic shocks: pathways toward integrated assessment. Earth’s Future.

[41] Moser SC, FinziHart JA (2015). The long arm of climate change: societal teleconnections and the future of climate change impacts studies. Clim. Change.

[42] Ryan, G. Climate of Santa Barbara, California. NOAA Technical Memorandum, NWS WR-225, U.S. Department of Commerce (1994).

[43] Dettinger MD, Ralph FM, Das T, Neiman PJ, Cayan DR (2011). Atmospheric rivers, floods and the water resources of California. Water.

[44] Barnard PL (2015). Coastal vulnerability across the Pacific dominated by El Niño/Southern oscillation. Nat. Geosci..

[45] Barnard PL (2017). Extreme oceanographic forcing and coastal response due to the 2015–2016 El Niño. Nat. Commun..

[46] Myers MR (2019). A multidisciplinary coastal vulnerability assessment for local government focused on ecosystems, Santa Barbara area. California. Ocean Coast. Manag..

[47] Pierce, D. W., Cayan, D. R. & Kalansky, J. F. Climate, drought, and sea level rise scenarios for the Fourth California Climate Assessment. California’s Fourth Climate Change Assessment. Publication #CCCA4-CEC-2018-006, California Energy Commission (2018).

[48] Barnard PL (2014). Development of the Coastal Storm Modeling System (CoSMoS) for predicting the impact of storms on high-energy, active-margin coasts. Nat. Hazards.

[49] Barnard PL (2019). Dynamic flood modeling essential to assess the coastal impacts of climate change. Sci. Rep..

[50] Feng D (2019). Propagation of future climate conditions into hydrologic response from coastal southern California watersheds. Clim. Change.

[51] Dugan JE, Hubbard DM, Quigley BJ (2013). Beyond beach width: steps toward identifying and integrating ecological envelopes with geomorphic features and datums for sandy beach ecosystems. Geomorphology.

[52] Jones, J. M. *et al*., Community exposure in California to coastal flooding hazards enhanced by climate change, reference year 2010. U.S. Geological Survey data release. Deposited 1 July 2016.

[53] Ganju NK (2017). Spatially integrative metrics reveal hidden vulnerability of microtidal salt marshes. Nat. Commun..

[54] Nowosad J, Stepinski TF (2019). Stochastic, empirically informed model of landscape dynamics and its application to deforestation scenarios. Geophys. Res. Lett..

[55] Intergovernmental Panel on Climate Change. Climate Change 2013: the Physical Science Basis. Contribution of Working Group I to the Fifth Assessment Report of the Intergovernmental Panel on Climate Change (2013).

[56] World Meteorological Organization, State of the Global Climate 2020. WMO-No. 1264 (2021).

[57] NASA Goddard Institute for Space Studies, GISS Surface Temperature Analysis (GISTEMP). https://data.giss.nasa.gov/gistemp/. Aaccessed 11 March 2020.

[58] National Research Council, Climate Change: Evidence and Causes: Update 2020. The National Academies Press, 36 pp. 10.17226/25733 (2020).

[59] Henley BJ, King AD, modulation by the Interdecadal Pacific Oscillation (2017). Trajectories toward the 1.5°C Paris target. Geophys. Res. Lett..

[60] Zedler, J. B., Nordby, C. S. & Kus, B. E. The ecology of Tijuana Estuary, California: a National Estuarine Research Reserve. NOAA Office of Coastal Resource Management, Sanctuaries and Reserves Division, Washington, D. C., 110 pp (1992).

[61] Thorne KG, Takekawa JY, Elliott-Fisk DL (2012). Ecological effects of climate change on salt marsh wildlife: a case study from a highly urbanized estuary. J. Coast. Res..

[62] Myers, M. R. *et al*. Santa Barbara Area Coastal Ecosystem Vulnerability Assessment. CASG-17–009. https://caseagrant.ucsd.edu/project/santa-barbara-area-coastal-ecosystem-vulnerability-assessment-sba-ceva (2017).

[63] Reynolds LC (2018). Coastal flooding and the 1861–2 California storm season. Mar. Geol..

[64] Duffy KA (2021). How close are we to the temperature tipping point of the terrestrial biosphere?. Sci. Adv..

[65] Clark PU (2016). Consequences of twenty-first-century policy for multi-millennial climate and sea-level change. Nat. Clim. Chang..

[66] Kulp SA, Strauss BH (2019). New elevation data triple estimates of global vulnerability to sea-level rise and coastal flooding. Nat. Commun..

[67] Dugan JE, Hubbard DM, Rodil IF, Revell DL, Schroeter S (2008). Ecological effects of coastal armoring on sandy beaches. Mar. Ecol..

[68] Jaramillo E, Dugan JE, Hubbard DM, Manzano M, Duarte C (2021). Ranking the ecological effects of coastal armoring on mobile invertebrates across intertidal zones on sandy beaches. Sci. Total Environ..

[69] Dugan JE, Hubbard DM, McCrary MD, Pierson MO (2003). The response of macrofauna communities and shorebirds to macrophyte wrack subsidies on exposed sandy beaches of Southern California. Estuar. Coast. Shelf Sci..

[70] Dugan JE, Hubbard DM, Page HM, Schimel JP (2011). Marine macrophyte wrack inputs and dissolved nutrients in beach sands. Estuar. Coasts.

[71] Hubbard DM, Dugan JE (2003). Shorebird use of an exposed sandy beach in southern California. Estuar. Coast Shelf Sci..

[72] Goodridge BM, Melack JM (2014). Radon residence times reveal temporal evolution and variability of dissolved inorganic nitrogen in beach pore water. Environ. Sci. Tech..

[73] Lowman HE, Emery KA, Kubler-Dudgeon L, Dugan JE, Melack JM (2019). Contribution of macroalgal wrack consumers to dissolved inorganic nitrogen concentrations in intertidal pore waters of sandy beaches. Estuar. Coast Shelf Sci..

[74] Hubbard DM, Dugan JE, Schooler NK, Viola S (2014). Local extirpations and regional declines: the case of endemic upper beach fauna in southern California. Estuar. Coast. Shelf Sci..

[75] Schooler NK, Dugan JE, Hubbard DM, Straughan D (2017). Local scale processes drive long-term change in biodiversity of sandy beach ecosystems. Ecol. Evol..

[76] Dugan JE, Hubbard DM (2010). Loss of coastal strand habitat in southern California: the role of beach grooming. Est. Coasts..

[77] Vitousek S, Barnard PL, Limber P, Erikson LH, Cole B (2017). A model integrating longshore and cross-shore processes for predicting long-term shoreline response to climate change. J. Geophys. Res. Earth.

[78] Griggs GB, Patch K (2019). The protection/hardening of California’s coast: times are changing. J. Coast. Res..

[79] Milne GA, Long AJ, Bassett SE (2005). Modelling Holocene relative sea-level observations from the Caribbean and South America. Quat. Sci. Rev..

[80] Stein, E. D. *et al.* Wetlands of the southern California coast: historical extent and change over time. Southern California Coastal Water Research Project, SCCWRP Technical Report No. 826, 50 pp. (2014).

[81] Grewell, B. J., Callaway, J. C. & Ferren, W. R. Jr. “Terrestrial vegetation of California” in Estuarine wetlands, 3rd edn, Barbour, M. G., Keeler-Wolf, T. & Schoenherr, A.A., Eds. (University of California Press, Los Angeles), pp. 124–154 (2007).

[82] Thorne KG (2018). US Pacific coastal wetland resilience and vulnerability to sea-level rise. Sci. Adv..

[83] Neijnens FK, Siteur K, van de Koppel J, Rietkerk M (2021). Early warning signals for rate-induced critical transitions in salt marsh ecosystems. Ecosystems.

[84] Griggs, G. B. & Russell, N. L. City of Santa Barbara: sea-level rise vulnerability study. california energy commission public interest energy research program report# CEC-500–2012–039, 89 pp. https://ww2.energy.ca.gov/2012publications/CEC-500-2012-039/CEC-500-2012-039.pdf (2012).

[85] National Research Council, Sea Level Rise for the Coasts of California, Oregon and Washington: Past, Present and Future (The National Academies Press, 2012), 216 pp. 10.17226/13389.

[86] Little Hoover Commission, Governing California Through Climate Change. Report #221. https://lhc.ca.gov/report/governing-california-through-climate-change (2014).

[87] State of California. Tsunami Hazard Area Map. California Geological Survey, California Governor’s Office of Emergency Services and AECOM. www.conservation.ca.gov/cgs/tsunami/maps (2021).

[88] Befus KM, Barnard PL, Hoover DJ, Finzi Hart JA, Voss CI (2020). Increasing threat of coastal groundwater hazards from sea-level rise in California. Nat. Clim. Change.

[89] Destination Analysts. 2016/17 Santa Barbara South Coast visitor profile and tourism economic impact study. 2018 report prepared for Visit Santa Barbara. https://santabarbaraca.com/wp-content/uploads/2017/10/Santa-Barbara-Visitor-Profile-and-Economic-Impact-Study-2016-17-DECK.pdf. Aaccessed 5 May 2021.

[90] King PG, Nelsen C, Dugan JE, Hubbard DM, Martin KL (2018). Valuing beach ecosystems in an age of retreat. Shore Beach.

[91] Taylor KE, Stouffer RJ, Meehl G (2012). An overview of CMIP5 and the experiment design. Bull. Am. Meteorol. Soc..

[92] Pierce DW, Cayan DR, Thrasher BL (2014). Statistical downscaling using localized constructed analogs (LOCA). J. Hydrometeorol..

[93] Cayan DR (2008). Climate change projections of sea level extremes along the California coast. Clim. Change.

[94] Erikson LH (2018). Projected 21st century coastal flooding in the Southern California Bight—Part 2: tools for assessing climate change driven coastal hazards and socio-economic impacts. J. Mar. Sci. Eng..

[95] O’Neill AC (2018). Projected 21st century coastal flooding in the Southern California Bight—part 1: development of the third generation CoSMoS model. J. Mar. Sci. Eng..

[96] Tolman HL (2002). Development and implementation of wind-generated ocean surface wave models at NCEP. Weather Forecast..

[97] Erikson LH, Hegermiller CA, Barnard PL, Ruggiero P, van Ormondt M (2015). Projected wave conditions in the Eastern North Pacific under the influence of two CMIP5 climate scenarios. Ocean Model.

[98] Booij N, Ris RC, Holthuijsen LH (1999). A third-generation wave model for coastal regions, part I: model description and validation. J. Geophys. Res..

[99] O’Neill AC, Erikson LH, Barnard PL (2017). Downscaling wind and wave fields for 21st century coastal flood hazard projections in a region of complex terrain. Earth Space Sci..

[100] Pierce, D. W. LOCA statistical downscaling (localized constructed analogs)—statistically downscaled CMIP5 climate projections for North America. Scripps Institution of Oceanography, http://loca.ucsd.edu/. Accessed 15 December 2017.

[101] Roelvink D (2009). Modelling storm impacts on beaches, dunes and barrier islands. Coast. Eng..

[102] Danielson JJ (2016). Topobathymetric elevation model development using a new methodology—Coastal National Elevation Database. J. Coastal Res..

[103] Erikson LH (2018). Identification of storm events and contiguous coastal sections for deterministic modeling of extreme coastal flood events in response to climate change. Coast. Eng..

[104] Limber P, Barnard PL, Vitousek S, Erikson LH (2018). A model ensemble for projecting multi-decadal coastal cliff retreat during the 21st century. J. Geophys. Res. Earth.

[105] Erikson, L. H., O’Neill, A. C., Barnard, P. L., Vitousek, S. & Limber, P. Climate change-driven cliff and beach evolution at decadal to centennial time scales. *Coastal Dynamics 2017* Paper No. 210, 125–136 (2017).

[106] Point Blue Conservation Science and U.S. Geological Survey. Our Coast Our Future (OCOF). Web application, Petaluma, California. www.ourcoastourfuture.org. Accessed 25 May 2021.

[107] Barnard, P. L. *et al*. Coastal storm modeling system (CoSMoS) for Southern California, v3.0, Phase 2 (ver. 1g, May 2018). U.S. Geological Survey data release. 10.5066/F7T151Q4. Deposited 13 May 2018.

[108] Moore LJ, Ruggiero P, List JH (2006). Comparing mean high water and high water line shorelines: should proxy-datum offsets be incorporated into shoreline change analysis?. J. Coast. Res..

[109] Ruggiero P, List JH (2009). Improving accuracy and statistical reliability of shoreline position and change rate estimates. J. Coast. Res..

[110] Barnard, P. L. *et al*. Coastal Processes Study of Santa Barbara and Ventura County, California. U.S. Geological Survey Open-File Report 2009–1029. http://pubs.usgs.gov/of/2009/1029/ (2009).

[111] National Oceanic and Atmospheric Administration (NOAA). Tides and currents, Cent. For Oper. Prod. And Serv., Silver Spring, MD. http://tidesandcurrents.noaa.gov/. Accessed 15 Feb 2020.

[112] Jones JM, Henry K, Wood N, Jamieson M (2017). HERA: a dynamic web application for visualizing community exposure to flood hazards based on storm and sea level rise scenarios. Comput. Geosci..

[113] U.S. Census Bureau, Explore Census Data. https://data.census.gov/cedsci/. Accessed 3 March 2020.

[114] Data Axle, Home page. https://www.data-axle.com/. Accessed 3 March 2020.

[115] U.S. Department of Homeland Security, Homeland Infrastructure Foundation-Level Data. https://gii.dhs.gov/hifld/. Accessed 3 March 2020.

[116] Multi-Resolution Land Characteristics Consortium, 2016 National Land Cover Database. https://www.mrlc.gov/data. Accessed 3 March 2020.

[117] Wood, N., Jones, J., Henry, K., Ng, P. & Hou, C. Y. Hazard exposure reporting and analytics, U.S. Geological Survey web application. https://www.usgs.gov/apps/hera. Accessed 3 March 2021.

